# The aged lymphoid tissue environment fails to support naïve T cell homeostasis

**DOI:** 10.1038/srep30842

**Published:** 2016-08-02

**Authors:** Bryan R. Becklund, Jared F. Purton, Chris Ramsey, Stéphanie Favre, Tobias K. Vogt, Christopher E. Martin, Darina S. Spasova, Gor Sarkisyan, Eric LeRoy, Joyce T. Tan, Heidi Wahlus, Brea Bondi-Boyd, Sanjiv A. Luther, Charles D. Surh

**Affiliations:** 1 Department of Developmental Immunology, La Jolla Institute for Allergy and Immunology, La Jolla, CA, 92037, USA; 2 Department of Immunology and Microbial Science, The Scripps Research Institute, La Jolla, CA, 92037, USA; 3 Department of Biochemistry, Center for Immunity and Infection, University of Lausanne, 1066 Epalinges, Switzerland; 4 Academy of Immunology and Microbiology, Institute for Basic Science, Pohang, Republic of Korea; 5 Department of Integrative Biosciences and Biotechnology, Pohang University of Science and Technology, Pohang, Republic of Korea

## Abstract

Aging is associated with a gradual loss of naïve T cells and a reciprocal increase in the proportion of memory T cells. While reduced thymic output is important, age-dependent changes in factors supporting naïve T cells homeostasis may also be involved. Indeed, we noted a dramatic decrease in the ability of aged mice to support survival and homeostatic proliferation of naïve T cells. The defect was not due to a reduction in IL-7 expression, but from a combination of changes in the secondary lymphoid environment that impaired naïve T cell entry and access to key survival factors. We observed an age-related shift in the expression of homing chemokines and structural deterioration of the stromal network in T cell zones. Treatment with IL-7/mAb complexes can restore naïve T cell homeostatic proliferation in aged mice. Our data suggests that homeostatic mechanisms that support the naïve T cell pool deteriorate with age.

Aging leads to a gradual functional decline in both the innate and adaptive arms of the immune system and is correlated with higher morbidity and mortality rates in the elderly in response to infectious diseases. Additionally, vaccine efficacy is reduced in elderly individuals rendering them more susceptible to common infections^1^. For example, influenza vaccination is only 17–53% efficacious in the elderly compared to 70–90% efficacy in young adults^2^. A major factor contributing to age-related defects in immunological responses is the progressive deterioration of naïve T cell function, including reduced expansion upon activation, decreased cytokine production, inefficient B cell help, and production of a defective memory T cell population^3^. The decline of immunological function is further amplified by a reduction in the diversity of the naïve T cell repertoire with aging^4^. Collectively, these defects diminish the ability of T cells to properly perform effector functions leading to suboptimal cell-mediated immune responses in aged individuals.

One of the hallmarks of aging in the immune system of mice and humans is the progressive shift in the T cell population from a predominantly naïve phenotype during youth to mainly memory phenotype in the elderly^5^,^6^. The prevailing view has been that the age-dependent memory phenotype shift is primarily driven by exposure to a lifetime of environmental antigens and reduced output of naïve T cells due to thymic involution. However, the thymus continues to produce low numbers of naïve T cells^7^,^8^ and the TCR diversity of the naïve T cell pool is maintained long after thymic involution^9^. Moreover, naïve T cells have a long lifespan as long as they receive the necessary survival signals. Thus, other mechanisms are likely involved in promoting the phenotypic shift with aging.

Naïve T cell survival in the periphery is reliant on entry into the secondary lymphoid organs (SLO) where they receive homeostatic signals essential for their survival^10^,^11^. Recruitment into the SLO is dependent on interactions between the chemokines CCL19 and CCL21 and their receptor CCR7 as well as other adhesion molecules. Movement through the SLO is aided by interactions with a complex network of supporting stromal cells including fibroblastic reticular cells (FRC) in T cell zones and follicular dendritic cells (FDC) in B cell zones. Stromal cells provide an architectural framework that compartmentalizes the SLO into discreet T and B cell zones and also play a more active role in mediating T cell survival; hence, FRC have been shown to be a primary source of IL-7, which is essential for T cell survival^11^,^12^. Naïve T cells are also dependent on low-level TCR stimulation through contact with antigen presenting cells (APC) bearing self-peptide MHC complexes within the SLO. The same factors that promote survival can also drive naïve T cell homeostatic proliferation and differentiation into memory phenotype under lymphopenic conditions^12^,^13^,^14^. Thus, competition for these survival factors helps maintain the overall naïve T cell population size and diversity in the periphery. We reasoned that perturbations in this system with aging could compromise naïve T cell survival and play a role in skewing the T cell pool toward a memory phenotype.

To address this possibility, we compared the ability of young and aged mice to support homeostasis of naïve T cells. Our results indicate that naïve T cell survival and homeostatic proliferation was compromised in aged mice. Surprisingly, the defect was not simply due to decreased levels of IL-7 with aging, but rather due to age-related changes in the SLO environment that limited T cell access to essential survival factors. Our study suggests that the reduced output of naïve T cells caused by thymic involution with aging is further compounded by a secondary lymphoid tissue environment that is unable to fully support homeostasis of naïve T cells.

## Results

### Homeostatic proliferation of naïve T cells is impaired in aged hosts

**Figure 1:**
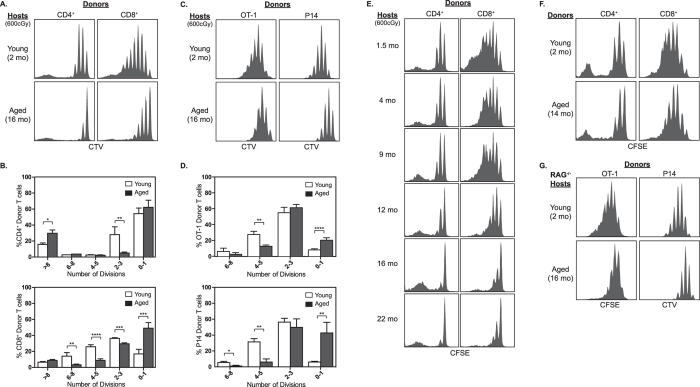
Reduced homeostatic proliferation of naïve T cells in aged mice. (**A**–**D**) Lymphocytes were isolated from young B6.CD45.1^+^, CD8^+^ OT-1, or CD8^+^ P14 LN, labeled with the proliferative dye CTV or CFSE, and 1.0–2.0 × 10^6^ cells were transferred into irradiated young or aged B6.CD45.2^+^ hosts. Proliferation of donor T cells in the LN of recipient mice was analyzed 7 days later by flow cytometry. Histograms show representative proliferation plots gated on donor polyclonal (**A**) or TCR transgenic (**C**) T cells. Bars represent the average percent of total donor polyclonal (**B**) or TCR transgenic (**D**) T cells that underwent the indicated number of divisions (±SD). (**E**) Representative histograms are shown depicting the proliferation of CFSE labeled B6.CD90.1^+^ donor T cells transferred into irradiated B6.CD90.2^+^ hosts of various ages 7 days earlier. (**F**) Histograms show the proliferation of donor T cells from either young or aged B6.CD45.1^+^ mice transferred into young (2 month) irradiated B6.CD45.2^+^ hosts. (**G**) Lymphocytes from young CD90.1^+^ CD8^+^ OT-1 or P14 donor mice were adoptively transferred into young or aged CD90.2^+^ RAG^−/−^ hosts. Representative histograms show proliferation of donor T cells 7 days after transfer. Experimental groups consisted of 2–3 mice per group and all experiments were repeated a minimum of 2 times. *P < 0.05, **P < 0.01, ***P < 0.001, ****P < 0.0001 as determined by unpaired Student’s T-test.

Previous reports have shown that the proportion of naïve CD4^+^ and CD8^+^ T cells in the SLO of aged mice is reduced and accompanied by a concomitant increase in the proportion of memory phenotype (MP) T cells^5^,^6^ (Figure S1A). The loss of naïve T cells is particularly dramatic in the peripheral lymph nodes (pLN) of aged mice^15^ (Figure S1B,C). To elucidate whether changes in the regulation of naïve T cell homeostasis contribute to the age-dependent decline in naïve T cell numbers, we measured the ability of aged hosts to support homeostatic proliferation, which is a highly sensitive method to monitor factors controlling naïve T cell survival. Lymphocytes were isolated from young donor mice, labeled with the proliferative dye CellTrace Violet (CTV), and adoptively transferred into young or aged CD45- or CD90-congenic hosts rendered lymphopenic by sublethal irradiation. Proliferation of donor cells was analyzed in the LN and spleen one week later. As previously shown for CD8^+^ T cells^16^, we found polyclonal B6 donor CD4^+^ and CD8^+^ T cells both showed a marked reduction in their ability to undergo homeostatic proliferation in aged hosts compared to young hosts (Fig. 1A,B). This finding also extended to the CD8^+^ T cell OT-1 and P14 TCR transgenic lines (Fig. 1C,D). To determine the relative age when the hosts’ ability to support efficient homeostatic proliferation declines, CFSE labeled lymphocytes from young B6 donor mice were transferred into irradiated B6 hosts of various ages. Defects in homeostatic proliferation became evident at 12 months of age and progressed to a nadir around 16 months of age (Fig. 1E). Finally, to determine whether defects in homeostatic proliferation were equally present in the SLO, aged B6 hosts were injected with FTY720 after adoptive transfer of donor T cells. Decreased proliferation of donor B6 T cells occurred equally in pLN, mLN and spleen (Figure S2A,B) indicating that aged mice exhibited a reduced ability to support homeostatic proliferation throughout the SLO.

### The aged host environment fails to support homeostatic proliferation of naïve T cells

It is well established that naïve T cells from aged individuals harbor a number of T cell intrinsic defects rendering them less responsive to foreign antigen stimulation^17^. Many of the same signaling pathways involved in responses to foreign antigens are thought to be necessary for efficient homeostatic proliferation to occur. Hence, to determine whether aging induces T cell intrinsic defects in homeostatic proliferation, LN cells from young (91% naïve among T cells) and aged (80% naïve among T cells) B6 mice were transferred into young irradiated B6 hosts. Interestingly, aged B6 donor T cells were only slightly impaired in their ability to undergo homeostatic proliferation compared to young B6 donor T cells (Fig. 1F). Thus, the age-dependent reduction in the number of naïve T cells appears to be primarily due to the aged host environment and not the aged T cell itself. One possible reason for the reduced ability of aged hosts to support homeostatic proliferation of naïve T cells could be due to increased competition for homeostatic factors from the expanded pool of MP T cells present in aged mice. To test this idea CD8^+^ OT-1 or P14 TCR transgenic cells in a RAG-1^−/−^ background were injected into young and old RAG^−/−^ deficient mice, which lack T cells. Notably, proliferation of CD8^+^ OT-1 and P14 donor T cells was dramatically reduced in old RAG^−/−^ hosts compared to young RAG^−/−^ hosts (Fig. 1G) indicating that competition from MP T cells is not responsible for the decrease in homeostatic proliferation in aged hosts.

### Memory T cell homeostatic proliferation is compromised in aged mice

**Figure 2:**
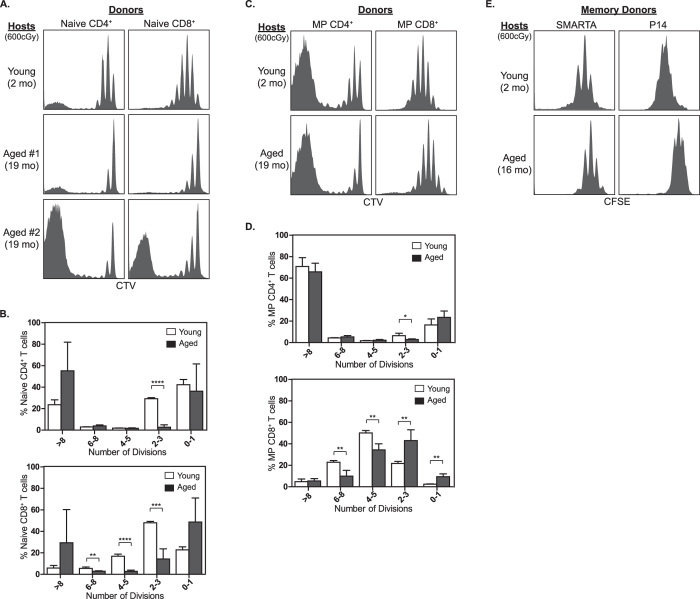
Impaired homeostatic proliferation of naïve and memory T cells in aged hosts. (**A–D**) Lymphocytes were isolated from the LN and spleen of young B6.CD90.1^+^ Foxp3^GFP+^donor mice. Naïve (CD44^low^ Foxp3^GFP−^) and memory phenotype (CD44^high^ Foxp3^GFP−^) CD4^+^ and CD8^+^ T cells were sorted by FACS, labeled with CTV, and 2.0 × 10^6^ cells were adoptively transferred into irradiated (600 cGy) B6.CD90.2^+^ young or aged hosts. Eight days later, proliferation of donor cells was analyzed by FACS in the LN and spleen of host mice. Histograms are shown demonstrating the proliferation of naïve (**A**) or MP (**C**) T cells. Bars represent the average percent of naïve (**B**) or MP (**D**) donor T cells that underwent the indicated number of cell divisions (±SD). (**E**) Small numbers (10^3^) of naïve TCR transgenic CD4^+^ Smarta and CD8^+^ P14 CD90.1^+^ T cells were injected into B6.CD90.2^+^ hosts and the host mice were infected with LCMV. Six weeks after infection Smarta and P14 memory T cells were isolated, labeled with CFSE, and injected into irradiated young or aged B6.CD90.2^+^ hosts. Proliferation was analyzed 7 days later by FACS. Histograms show representative CFSE profiles for donor T cells. Each experiment consisted of 2–3 mice per group and was repeated a minimum of 2 times. *P < 0.05, **P < 0.01, ***P < 0.001, ****P < 0.0001 as determined by unpaired Student’s T-test.

Although the experiments described above demonstrate a deficit in the ability of aged hosts to support naïve T cell homeostatic proliferation, it was not clear whether memory T cell homeostasis was also altered in the aged environment. To test this notion, sorted young CTV-labeled naïve (CD44^low^ Foxp3^GFP−^) and MP (CD44^high^ Foxp3^GFP−^) B6 T cells were injected into irradiated young and aged B6 hosts. As expected, analysis of naïve donor T cell proliferation after 8 days revealed a clear impairment of homeostatic proliferation in aged hosts compared to young hosts (Fig. 2A,B) (Aged #1). Surprisingly, a fraction of naïve T cells underwent a prodigious rate of proliferation in ~50% of the aged hosts that we analyzed rendering the majority of donor T cells, especially CD4^+^ T cells, to be CTV^−^ (Fig. 2A,B) (Aged #2). This finding resembles the behavior of polyclonal naïve T cells transferred into chronically immunodeficient hosts, such as RAG^−/−^ mice, where a fraction of T cells responds strongly to enteric antigens derived from the commensal microbiota^18^. Future experiments will address whether dysregulation in presentation of commensal bacterial and/or dietary antigens is driving rapid proliferation of a subset of naïve T cells in aged hosts.

Impaired homeostatic proliferation was also noted in B6 MP donor T cells transferred into irradiated aged hosts (Fig. 2C,D). MP CD8^+^ T cells, which proliferated at a faster rate than their naïve CD8^+^ counterparts, underwent a noticeably slower rate of proliferation in aged hosts. However, the reduction was less dramatic than that observed with B6 naïve CD8^+^ T cells in aged hosts. MP CD4^+^ T cells produced a heterogeneous proliferation profile comprised of distinct slowly and rapidly dividing populations (Fig. 2C,D). Previous studies have demonstrated that the slowly dividing MP CD4^+^ T cells are responding primarily to cytokines, whereas the fast dividing population is dependent on TCR interactions with class-II MHC^19^. In irradiated aged hosts there was a noticeable reduction in cytokine-dependent slow proliferation compared to young hosts, but MHC-II-dependent fast proliferation was largely unaffected.

Previous studies have shown that the homeostatic requirements differ between the heterogeneous population of MP and Ag-specific memory T cells raised from intentional immunization to a foreign antigen^19^. Therefore, we sought to determine whether the age-specific deficits in homeostatic proliferation of polyclonal B6 MP CD4^+^ and CD8^+^ T cells also occurred for Ag-specific memory T cells. To this end, memory CD4^+^ Smarta and CD8^+^ P14 TCR transgenic T cells generated using young mice were found to undergo a slower rate of homeostatic proliferation in aged hosts compared to young hosts (Fig. 2E). Thus, aging leads to decreased homeostatic proliferation of both polyclonal MP and Ag-specific memory T cells. However, the age-dependent deficit in homeostatic proliferation was less pronounced for memory T cells compared to naïve T cells.

### Naïve T cell survival is decreased in aged mice

**Figure 3:**
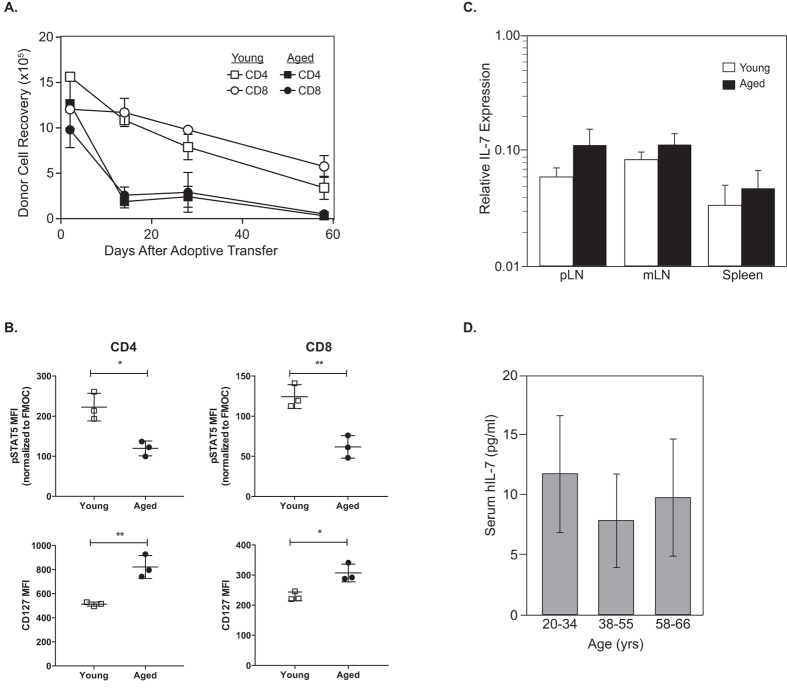
Despite normal IL-7 levels, naïve T cell survival is decreased in aged mice. (**A**) Groups of non-irradiated young (2 months) and aged (18 months) B6.CD45.2^+^ mice were injected with 12 × 10^6^ CFSE labeled whole LN cells from young B6.CD45.1^+^ donor mice. Donor T cell recovery from the LN and spleen of host mice was determined at various time points. (**A**) Each time point depicts the average recovery of undivided (CFSE^high^) naïve T cells (±SD) from 2–3 host mice per group. (**B**) Naïve T cells from young B6.CD45.1^+^ mice were transferred into irradiated young and aged B6.CD45.2^+^ hosts. Donor cell STAT5 phosphorylation normalized to a fluorescence minus one control (FMOC) and CD127 expression in the pLN was determined one day later by flow cytometry. Data are representative of 2 independent experiments with 2–3 mice per group. *P < 0.05 and **P < 0.01 as determined by unpaired Student’s T-test. (**C**) Total mRNA was isolated from the pLN, mLN, and spleen of young (2 month) and aged (24 month) mice. Bars show the average normalized IL-7 mRNA expression (±SD) as determined by qPCR. Samples were run in triplicate with 2–3 mice per group. (**D**) Bars depict the concentration of IL-7 protein (±SD) as determined by ELISA in the serum of 28 healthy human volunteers from 20–66 years of age. The samples were analyzed in triplicate and split into three arbitrary age groups with 9–10 individual samples per group.

Since factors that drive homeostatic proliferation in lymphopenic hosts are also critical for T cell survival under normal conditions, we sought to determine whether the aged host environment is also impaired in its ability to support survival of naïve T cells under normal physiological conditions. To this end, non-irradiated young and aged B6 mice were injected with CFSE-labeled lymphocytes from young B6 mice and recovery of CFSE^hi^ (undivided) naïve donor T cells in host LN and spleen was assessed at various time-points. Donor cell recovery one day after adoptive transfer was used as the initial graft reference point. In agreement with a previous report^20^, donor CD4^+^ and CD8^+^ T cells underwent a slow, gradual loss with a half-life of approximately 4 weeks in young hosts (Fig. 3A). In contrast, a rapid loss of the majority (75–85%) of donor CD4^+^ and CD8^+^ T cells occurred within the first 2 weeks in aged hosts with the remaining cells disappearing gradually over the next 6 weeks (Fig. 3A). The dramatic accelerated decrease in T cell recovery in aged hosts indicated that naïve T cells failed to receive survival signals in the aged environment.

### IL-7 signaling is impaired in aged mice

The cytokine IL-7 plays a central role in maintaining naïve T cell survival in the periphery^11^,^12^. IL-7 signals through a functional IL-7 receptor (IL-7R) composed of a unique α-chain (CD127) bound to the common γ chain (CD132). Signaling through the IL-7R leads to phosphorylation of the signal transducer and activator of transcription 5 (STAT5) and regulation of target genes including a decrease in CD127 expression^21^,^22^. To determine whether IL-7-induced signaling was altered in aged mice, B6 naïve or MP T cells were transferred into irradiated young and aged B6 hosts and then the phosphorylation state of the STAT5 and expression levels of CD127 on donor T cells analyzed 1 day later. Naïve donor CD4^+^ and CD8^+^ T cells had significantly lower STAT5 phosphorylation with higher levels of CD127 expression in aged hosts compared to young hosts (Figs 3B and S3A,B). Although there was a trend for reduced pSTAT5 and increased CD127 levels in MP CD4^+^ and CD8^+^ donor cells, the difference was not significant (S3C). Collectively, these findings indicate that naïve T cells in aged mice receive suboptimal signaling through the IL-7R, whereas this signaling deficiency appears to be partially compensated in memory T cells by related cytokines, such as IL-15.

### IL-7 expression does not vary significantly with age

The simplest possible explanation for the defects in IL-7 signaling in aged mice is due to a reduction in IL-7 expression. To test this notion, IL-7 mRNA was quantified in the SLO of young and aged B6 mice using quantitative PCR. Surprisingly, we did not find any significant difference in the expression of IL-7 mRNA in any of the tissues tested (Fig. 3C). Quantifying IL-7 protein in mice is hampered by the lack of a sensitive enough ELISA, but this approach is possible with humans. Hence, the amount of IL-7 protein in human serum from healthy volunteers ranging from 20 to 66 years old was analyzed by ELISA. In agreement with our mouse data and a previous publication^23^, the concentration of IL-7 was similar in the different age groups (Fig. 3D). Combined, these results indicate that the defects in naïve T cell homeostasis in the aged are not simply caused by an age-dependent decrease in production of IL-7.

### Naïve T cell recruitment and chemokine expression are altered in aged SLO

**Figure 4:**
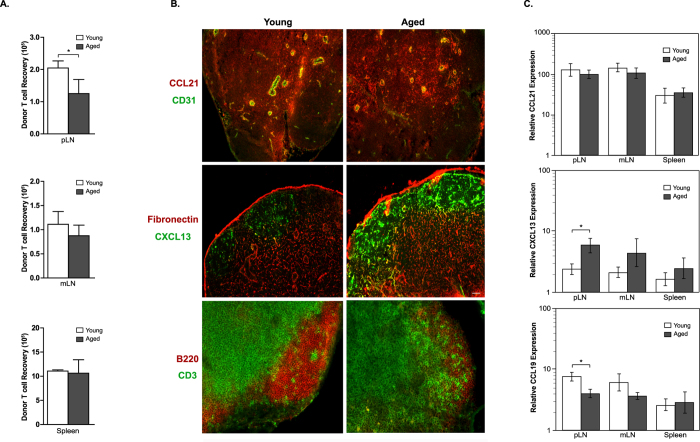
Dysregulated chemokine expression is associated with diminished T cell homing to aged LNs. (**A**) Lymphocytes were isolated from B6.CD90.1^+^ Foxp3^GFP+^ donor mice and 8.0 × 10^6^ cells were injected into young (2 month) and aged (16 month) B6.CD90.2^+^ hosts. Bars show naïve donor T cell recovery (CD44^low^, Foxp3 ^GFP−^) in the pLN, mLN, and spleen of young and aged mice 4 hours after adoptive transfer. (**B**) Representative images are shown from young and aged pLN sections that were stained with the indicated antibodies and analyzed using immunofluorescence microscopy. Scale bars equal 50 μm. (**C**) qPCR was used to determine the average normalized mRNA expression (±SD) of CCL21, CXCL13, and CCL19 in the pLN, mLN, and spleen of young and aged mice. *P < 0.05 as determined by unpaired Student’s T-test.

Naïve T cell survival and homeostatic proliferation requires T cell migration into the proper microenvironment of the SLO to gain access to homeostatic signals. Since IL-7 expression did not decline with age, we reasoned that aging might lead to changes in the SLO microenvironment. To this end, we first analyzed homing of naïve T cells into secondary lymphoid tissues in aged hosts by adoptively transferring B6 naïve T cells into young and aged B6 recipients and determining the number of donor cells in host SLO 4 hours later. We observed a significant decrease in the homing of naïve T cells to the pLN of aged hosts, while entry into the mLN and spleen was less affected (Fig. 4A).

Naïve T cell entry from the blood into SLO is mediated in part by interactions between CCR7 expressed on T cells with the chemokines CCL19 and CCL21 on the surface of high endothelial venules (HEVs). Moreover, selective expression of CCL19 and CCL21 by fibroblastic reticular cells (FRCs) in the T cell zone, along with CXCL13 expression by follicular dendritic cells (FDCs) and marginal reticular cells (MRCs) in the follicles, control the migration and compartmentalization of lymphocytes into their respective T and B cell zones. To determine if changes in chemokine expression could explain the age-dependent defect in naïve T cell recruitment to SLO, young and aged B6 LN sections were stained for CCL21 and the endothelial cell marker CD31 to visualize HEVs. We failed to see a significant difference in the expression of CCL21 protein (Fig. 4B-top) or mRNA (Fig. 4C-top) in the SLO of young and aged mice. The number of HEVs was also similar in young and aged LN, but they appeared more dense and compressed in the aged LN (Fig. 4B-top). Unfortunately, we and others were unable to detect CCL19 protein by immunofluorescence staining^11^,^24^. However, we found that CCL19 mRNA levels were reduced in the pLN of aged B6 mice (Fig. 4C-bottom). Formation of follicles is dependent on CXCL13 to recruit and maintain CXCR5-expressing B cells into B cell zones. In agreement with previous findings, we noted a dramatic increase in the expression of CXCL13 protein (Fig. 4B-middle) and mRNA (Fig. 4C-middle) in the pLN of aged B6 mice^25^. To determine if defects in chemokine expression in aged LN were associated with altered compartmentalization, we stained young and aged B6 LN to identify CD3^+^ T cells in T cell zones and B220^+^ B cells in the follicles. As expected, immunofluorescence staining revealed that young LN were separated into discreet T and B cell zones with a few T cells present in the follicles (Fig. 4B-bottom). In half of the LN from aged mice this segregation pattern was altered with a large number of CD3^+^ T cells improperly segregated into B cell zones (Fig. 4B-bottom). Moreover, the density of CD3^+^ T cells was noticeably reduced in aged mice. Collectively, these results indicate that changes in chemokine expression with age may contribute to altered homing and segregation of naïve T cells in aged SLO, particularly in the pLN.

### The stromal network is altered with age limiting access to survival signals

**Figure 5:**
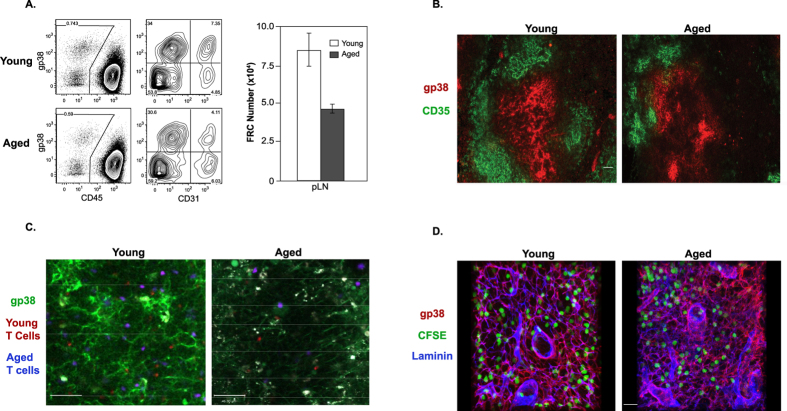
The stromal network is altered in the LN of aged mice. (**A**) LNs were removed from young and aged mice and FRCs were isolated by collagenase digestion. Plots show the gating strategy used to determine the total number of gp38^+^CD31^−^CD35^−^CD45^−^ FRCs. Graph depicts the average number of FRCs recovered from the pLN of young and aged mice. (**B**) Representative images are shown from young and aged spleen sections stained with anti-gp38 and CD35 antibodies and analyzed by immunofluorescence microscopy. Scale bars equal 50 μm. (**C**) LN cells from young (2 month) or aged (20 month) mice were labeled with CMTPX (red) or CMTMR (blue), respectively, and 5.0 × 10^6^ total cells were injected into age-matched littermate hosts. Hosts were injected with FITC-labeled anti-gp38 antibody 15–18 hours before analysis. Inguinal LN were explanted and visualized by multi-photon excitation microscopy. Images show representative data from 4 independent experiments and scale bars equal 50 μm. (**D**) Naive T cells were labeled with CFSE and transferred into young and aged hosts. Representative images are shown from young and aged pLN vibratome sections stained with anti-gp38 and laminin antibodies analyzed by immunofluorescence microscopy. Scale bars are equal to 20 μm.

Stromal cells such as FRCs and FDCs form a complex structural framework that facilitates the organization of immune cells into distinct microenvironments within the SLO. The central role that FRCs play in T cell trafficking and survival led us to investigate whether age-related changes in these cells could potentially explain the homeostatic defects noted in aged mice. To test this notion, the total number of FRCs (gp38^+^CD31^−^CD35^−^CD45^−^) was compared between young and aged B6 mice. Indeed, we noted a clear reduction in the number of FRCs recovered from the pLN of aged mice compared to young mice (Fig. 5A). Next, immunofluorescence staining was performed to identify both FRCs and CD35^+^ B zone FDCs. Both T and B zone stroma appeared compressed and less reticular in the aged than young B6 mice (Fig. 5B). Thus, not only were there fewer FRCs present in aged mice, but their structural network was abnormal.

To determine if FRC defects impaired lymphocyte access to the T cell zones of SLO, we transferred fluorescently-labeled whole LN cells from young or aged B6 mice into young and aged B6 hosts and analyzed donor cell movement by multi-photon excitation microscopy. Young and aged lymphocytes trafficked equally to the LN (Fig. 5C). However, the aged LN recruited fewer lymphocytes than the young LN and gp38 staining revealed disrupted FRC morphology in the aged hosts. Staining of vibratome sections of LN from young and old hosts from similar experiments also demonstrated that adoptively transferred donor naïve T cells were able to enter the pLN and appeared to interact with gp38^+^ FRCs in both hosts (Fig. 5D). However, the stromal network in aged LN appeared less organized, gp38 expression more diffused, and there were areas in the FRC network devoid of T cells (Fig. 5D). This is despite the finding that expression of extracellular matrix fibronectin (Fig. 4B) and laminin (Fig. 5D) appeared relatively normal without any obvious signs of fibrosis in aged pLN. These results indicate that the networks of FRC in the SLO degenerates with age and are impaired in their ability to recruit naïve T cells.

### IL-7/M25 antibody complexes can restore naïve T cell homeostatic proliferation in aged mice

**Figure 6:**
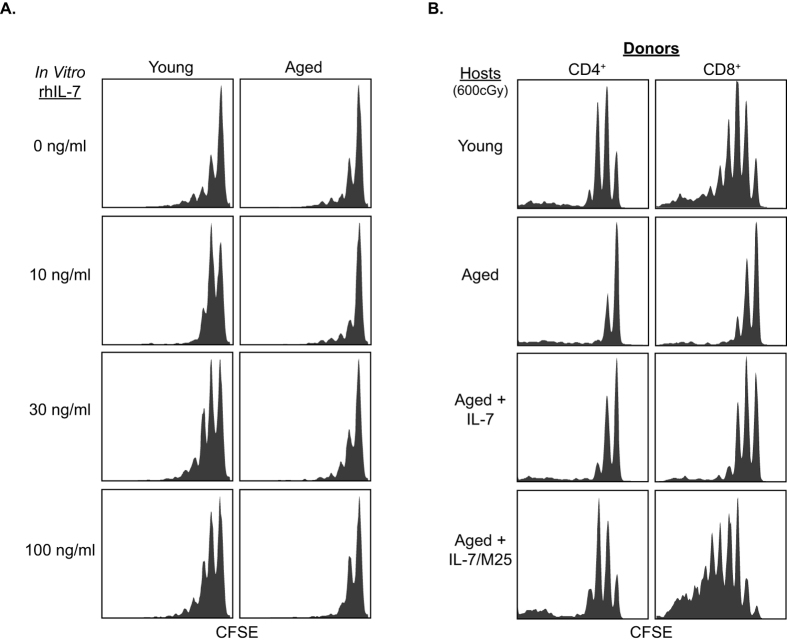
IL-7/M25 complexes can restore homeostatic proliferation in aged hosts, while IL-7 alone is insufficient. (**A**) Naïve CD90.1^+^ CD8^+^ OT-1 T cells were labeled with CFSE and injected into irradiated young (2 month) and aged (14 month) B6.CD90.2^+^ hosts. The LN were removed 1 day later and cultured *in vitro* for 7 days with varying amounts of exogenous IL-7. Proliferation of donor T cells was analyzed by FACS. Representative CFSE proliferation histograms are shown from one of 3 similar separate experiments with 2–3 mice per group. (**B**) Naïve T cells were sorted from CD90.1^+^ donor mice, labeled with CFSE, and injected into irradiated young (2 month) or aged (16 month) hosts. The host mice were injected every other day with either 1.5 μg of IL-7 alone or an equivalent IL-7 dose in a pre-formed complex with the IL-7 neutralizing antibody M25. Representative CFSE proliferation plots are shown from one of 3 independent experiments each with 2**–**3 mice per group.

Treatment with IL-7 is currently being explored as a potential method to restore the immunological defects associated with aging^26^. To determine whether delivering exogenous IL-7 could rescue the defects in naïve T cell homeostatic proliferation in aged hosts, we first utilized an *in vitro* LN organ culture system^20^. Hence, LN from irradiated young or aged B6 mice previously injected with CD8^+^ OT-1 cells were cultured at the air/liquid interphase supplemented with varying doses of IL-7. As expected, the addition of IL-7 to the media led to a dose-dependent increase in proliferation of donor OT-1 T cells in the LN of young mice (Fig. 6A). In contrast, exogenous IL-7 did not substantially increase donor OT-1 T cell proliferation in the LN of aged mice even at high doses (Fig. 6A). Thus, simply administering large amounts of IL-7 is unlikely to restore the ability of aged hosts to support naïve T cell homeostasis.

Finally, we tested whether administration of IL-7 can restore *in vivo* naïve T cell homeostatic proliferation in aged hosts. Based on above *in vitro* data, the effect of highly potent complexes of IL-7 bound to a neutralizing mAb M25^27^ was analyzed. Hence, irradiated young and aged B6 mice previously injected with CFSE-labeled B6 LN cells were injected every other day with either PBS, IL-7 alone, or IL-7/M25 antibody complexes. Strikingly, while administration of IL-7 alone had a minimal effect, treatment with IL-7/M25 complexes completely restored the age-dependent defects in homeostatic proliferation of donor CD4^+^ and CD8^+^ T cells in aged hosts (Fig. 6B).

## Discussion

The size and composition of the T cell pool is maintained by homeostatic mechanisms that govern naïve T cell survival in the periphery. Although the output of naïve T cells drops off dramatically following thymic involution, the overall size of the T cell pool remains remarkably stable throughout life. Thus, peripheral homeostatic mechanisms compensate for the declining production from the thymus by promoting naïve T cell survival and intermittent homeostatic proliferation to maintain the overall population size and diversity. Our current results indicate that these peripheral mechanisms begin to gradually deteriorate with age leading to an inhospitable environment for survival and renewal of naïve T cells.

In general, the age-associated defect in homeostatic proliferation was more pronounced for naïve T cells than memory T cells, likely due to differences in homeostatic requirements between the different cell types. Similar to naïve T cells, memory T cells depend on cytokines IL-7 and IL-15 for survival^12^. However, IL-15 plays a more prominent role in memory T cell homeostasis and memory T cells are less dependent on TCR interactions with self-peptide MHC molecules for survival and homeostatic proliferation. MP CD4^+^ T cells undergo two distinct patterns of proliferation^28^. Our data indicates that aging led to a reduction in the cytokine-dependent slow proliferation, but did not affect the MHCII-dependent rapid proliferation of MP CD4^+^ T cells. Collectively, these data suggest that memory T cell homeostasis is altered in aged hosts, but to a lesser extent, potentially favoring the survival of memory T cells over naïve T cells in the aged environment. Since memory T cells are primarily dependent on cytokine and not self-peptide MHC signaling, the defect in memory T cell homeostatic proliferation in aged mice suggests that diminished access to cytokines is primarily responsible for the age-dependent reduction in homeostatic proliferation of both naïve and memory T cells. In addition, the presence of naïve and memory CD4^+^ T cell populations that undergo TCR-driven homeostatic proliferation in the aged hosts could further contribute to depletion of naïve T cells.

The decline in naïve T cell survival and homeostatic proliferation does not appear to be due to intrinsic T cell defects or a decrease in the levels of IL-7 and IL-15. Although both cytokines are difficult to measure directly, we have shown that IL-7 mRNA levels do not change over time in peripheral lymphoid tissues of mice, nor do IL-7 protein levels change with age in the serum of human subjects. Moreover, previous studies have shown that IL-15 levels also remain stable with age^16^,^29^. Thus, the primary homeostatic defect appears to be an altered environment in aged lymphoid tissues which limits the ability of naïve T cells to access key survival factors, as manifested by decreased homeostatic proliferation, reduced STAT5 signaling and increased IL-7R upregulation. A potential caveat to this interpretation is that our methods lack the sensitivity to measure the IL-7 relevant for T cell homeostasis, which is thought to be produced by stromal cells in the T cell zones of secondary lymphoid tissues.

The impaired ability of naïve T cells to access survival signals in the aged lymphoid environment is likely due to a number of compounding defects. First, the ability of naïve T cells to home to lymphoid organs is reduced in aged mice. Naïve T cell entry into secondary lymphoid tissues is a multi-step process that is dependent in part on CCR7 interactions with the chemokines CCL19 and CCL21 expressed on the surface of HEVs. Furthermore, these chemokines in combination with CXCL13 mediate lymphocyte movement within the lymph node environment and play an important role in segregating T and B cells into their respective zones. We found no difference in the mRNA and protein expression of CCL21 with age. However, CCL19 mRNA levels were decreased in the LN, but not in the spleen of aged mice. A more dramatic difference was noted in CXCL13 expression, which was highly upregulated in aged mice, consistent with a previous study^25^. Interestingly, a previous study points to age-dependent changes in the lymphoid environment contributing to reduced T cell recruitment and dysregulated chemokine expression in response to infections^24^. Thus, age-dependent changes in the lymphoid environment may alter lymphocyte homing to the lymphoid tissues and limit access to environmental antigens as well as essential survival factors.

Another factor likely limiting naïve T cell access to survival factors is the altered stromal network found in aged secondary lymphoid tissues. Stromal cells, such as FRCs and FDCs, provide a structural framework and produce chemokines to help organize the overall architecture of the secondary lymphoid tissue. Moreover, they provide a cellular highway along which immune cells can move and interact with each other and homeostatic factors. Our results indicate that the stromal network is compromised in aged secondary lymphoid tissue. The number of FRCs recovered from pLN was reduced in aged mice. Moreover, FRC and FDC network was compressed and less reticular with larger gaps in gp38 staining in aged secondary lymphoid tissues. The consequences of this altered stromal network are not entirely clear and the frequency of anatomical disturbances varied in aged mice. One potential consequence is that the compressed stromal network may reduce the availability of homeostatic factors by spatially restricting access and/or by limiting T cell movement. Recent evidence indicates that coordinated signaling through cytokines and self-peptide MHC interactions are important for optimal naïve T cell survival^30^. Thus, stromal network defects may adversely alter spatiotemporally coordinated survival factor signaling in aged secondary lymphoid tissue.

Finally, the stromal network is also important for producing components of the extracellular matrix which are thought to play a prominent role in facilitating IL-7 presentation to T cells in the secondary lymphoid tissue. Although the extracellular matrix appeared normal, stromal defects in the aged lymphoid tissues may contribute to altered IL-7 presentation. In support of this, we found that treating aged mice with exogenous IL-7 failed to increase the *in vitro* or *in vivo* homeostatic proliferation of naïve donor T cells. Thus, the aged mice were unable to properly present exogenous IL-7. In contrast, administration of IL-7/M25 complex was able to restore homeostatic proliferation in aged mice. This finding indicates that IL-7/M25 complex can overcome the IL-7 accessibility and/or “presentation” defect apparent in aged hosts, presumably by extending the half-life of exogenous IL-7 and by preventing its rapid utilization^31^. Regardless of the mechanism, we have shown that age-dependent defects in supporting homeostatic proliferation can be reversed using IL-7/M25 complexes.

In closing, age-dependent changes in the SLO appear to limit access to key survival signals leading to decreased naïve T cell survival and homeostatic proliferation. The inability of the aged lymphoid tissue environment to support naïve T cell survival likely hastens the loss of an already diminished supply of naïve T cells due to thymic atrophy. Thus, gradual changes in factors regulating naïve T cell homeostasis may play an important role in defective immune responses in aged individuals by causing accelerated depletion of naïve T cells.

## Methods

### Mice

C57BL/6 (B6), B6.PL (B6.CD90.1^+^), B6.SJL (B6.CD45.1^+^), and B6.RAG-1^−/−^ mice were obtained from the breeding colony at The Scripps Research Institute (TSRI) or from The Jackson Laboratory. B6.Cg-Foxp3^tm2Tch^/J (B6.Foxp3^GFP^) mice, which coexpress enhanced GFP and Foxp3 under control of the endogenous Foxp3 promotor, were described previously^32^ and purchased from The Jackson Laboratory. Smarta^33^, P14^34^, and OT-1^35^ transgenic TCR mouse lines were described previously. Adult mice ranging from 2–4 months were included in the young group, while the aged group ranged from 14–26 months old mice. Mice were housed under specific pathogen free conditions at either TSRI, La Jolla Institute for Allergy and Immunology (LIAI) and Pohang University of Science and Technology (POSTECH). The Institutional Animal Care and Use Committees approved all animal protocols at the respective institutions. All methods were carried out in accordance with the approved guidelines.

### Adoptive transfer of lymphocytes

Inguinal, axillary, brachial, cervical and mesenteric LNs were collected and pooled from the indicated donor mice. A single cell suspension in DMEM supplemented with 0.5% FBS and 1.0% HEPES was made by gently homogenizing the LNs followed by straining through 100 μm mesh screen. In some experiments T cells were enriched by first staining with an antibody cocktail containing the following biotinylated antibodies purchased from Biolegend: B220, CD11b, CD11c, CD19, CD24, and NK1.1. The cells were then incubated with streptavidin-conjugated magnetic particles (BD Biosciences) and placed on a BD iMagnet to negatively enrich for T cells. Enriched T cells were stained with anti-CD4-APC, CD8-PB, and CD44-PE antibodies (Biolegend or eBioscience) and sorted into naïve (CD44^low^Foxp3^GFP−^) and MP (CD44^high^ Foxp3^GFP−^) fractions using a BD FacsAria cell sorter. Antigen specific memory cells were generated by transferring CD4^+^ Smarta and CD8^+^ P14 donor T cells from young adult mice into young adult B6 hosts and injecting with the LCMV Armstrong virus as described previously^19^. Antigen-specific memory cells were isolated 6 weeks after infection. Donor cells were labeled with either 5,6-carboxyfluoresein diacetate succinimidyl ester (CFSE) or CellTrace Violet (CTV) (both from Life Technologies) proliferation dyes to monitor cell turnover^36^. Recipient mice were irradiated with 600 cGy prior to the intravenous or retro-orbital injection of 1–2 × 10^6^ labeled donor T cells. In some experiments, lymph node egress was inhibited by i.p. injecting mice on days 1 and 3 after cell transfer with 3 mg/kg FT7-720 (Cayman Chemical). Proliferation of donor T cells was analyzed by flow cytometry 7–8 days after adoptive transfer. In some experiments, 8–12 × 10^6^ donor T cells were transferred to non-irradiated hosts and donor cell recovery was determined at various time-points.

### Antibodies and flow cytometry

Single cell suspensions derived from pLN, mLN, and spleen of host mice were stained with the indicated antibodies in PBS containing 0.5% FBS and 0.2% sodium azide. The following antibodies conjugated to various fluorophores were used for analysis: CD4 (RM4-5), CD8 (53-6.7), CD31 (MEC13.3), CD35 (7E9), gp38 (8.1.1), CD44 (IM7), Vα2 (B20.1), CD45.1 (A20), CD127 (A7R34) and CD90.1 (OX-7). Donor T cells were identified using anti-CD45.1 and CD90.1 antibodies. Anti-Vα2 was included to identify TCR transgenic donor T cells. Non-specific binding was blocked using 2.4G2 and propidium iodide was added to eliminate dead cells from the analysis. Intracellular staining for phosphorylated STAT5 was performed by fixing isolated cells with BD Fix Buffer I for 10 minutes at 37 °C followed by permeabilization in 90% methanol for 1 hour at 4 °C. After washing, cells were labeled overnight at 4 °C with a pY694-STAT5-PE antibody (BD biosciences). Isolation and staining of stromal cells was performed as previously described^11^. Data from stained cells was collected using a BD LSR-II flow cytometer.

### *In vitro* lymph node organ culture

Donor LN cells were labeled with CFSE and 2–5 × 10^6^ cells were injected into irradiated hosts. One day later, LNs were excised from the host mice and the organs were cultured at 37 °C in a 93% O_2_/ 7% CO_2_ atmosphere in DMEM media supplemented with 10% γ-globulin free horse serum, glutamine, penicillin/streptomycin, and neomycin as previously described^20^. Recombinant human IL-7 (NCI Biological Resources Branch) was delivered in the media at the indicated doses and the culture media was changed every other day. Single cell suspensions were made from the cultured LNs and stained with the appropriate antibodies followed by analysis by FACS.

### IL-7 Antibody Complexes

Recombinant human IL-7 (1.5 μg) was incubated for 30 minutes with 15 μg of the neutralizing anti-IL-7 antibody M25 to form a pre-bound complex. Mice were injected with either IL-7 alone or IL-7/M25 complexes delivering an equivalent IL-7 dose every other day for one week as described previously^27^.

### IL-7 ELISA

To determine IL-7 protein levels in human serum, 10 ml blood samples were collected from 28 healthy individuals ranging from 20–66 years of age. Blood samples were layered on Histopaque-1077 (Sigma) and serum was collected for analysis. Human IL-7 concentration was determined in 200 μl of serum using a commercially available ELISA kit following the manufacturer’s instructions (R&D). These experiments were done in compliance with TSRI institutional human subject protocols.

### Quantatative PCR

RNA was extracted using TRIzol reagent (Invitrogen). First-strand cDNA synthesis (Superscript II; Invitrogen) was performed according to the manufacturer’s instructions using random nonamer primers (Microsynth). cDNA was purified with the NucleoSpin Extract II Kit (Macherey-Nagel) and individual transcripts assessed by quantitative PCR using the Light Cycler-FastStart DNAMaster SYBR Green I kit (Roche Diagnostics) on a Light Cycler 2.0 machine (Roche Diagnostics). Efficiency-corrected gene expression of target genes was normalized with the geometric mean of expression of two housekeeping genes, hypoxanthine guanine phosphoribosyl transferase (*hprt1*) and TATA-binding protein (*tbp*).

### Immunofuorescence microscopy

Cryostat sections (8–10 μm) of Tissue-Tek OCT (Sakura)-embedded LN were collected on Superfrost/Plus glass slides (Fisher Scientific) then air dried overnight, fixed in ice-cold acetone for 10 min and rehydrated in PBS. Sections were quenched using 0.3% H_2_O_2_ in PBS, blocked using 0.1% BSA and 1–4% animal serum in PBS followed by a streptavidin-biotin blocking kit (Vector Laboratories), as described before^11^. Staining was performed for 60 min at room temperature using the indicated antibodies. For gp38, the staining was revealed using HRP-conjugated secondary reagents followed by Tyramide Signal Amplification (Molecular Probes Kit #22) according to the manufacturer’s instructions, but using a borate buffer (0.1 M in PBS, pH 8.5) for tyramide dilution. Images were acquired on Leica DM5500 microscope with a Leica DFC320 camera or on a DM IRE2 microscope with laser scanning confocal head TCS SP2 AOBS (Leica).

For vibratome sections, isolated LN were fixed overnight at 4 °C in freshly prepared 1% PFA in PBS, washed, embedded in 4% low-gelling agarose (Sigma) in PBS and 100–200 μm sections cut using a vibratome (Microm HM 650V). Sections were blocked with 1% BSA for 1 h and stained for at least 3–12 h with the indicated antibodies. Subsequently, sections were washed extensively in PBS and embedded using Elvanol (Mowiol, Calbiochem). Images were taken with a Zeiss Axio Imager upright microscope. 3D-image reconstructions were made with Imaris software and segment length was assessed using the FilamentTracer plugin (Bitplane).

### Multi-Photon Excitation Microscopy

Lymphocytes were isolated from young and aged donor mice and labeled with the fluorescent dyes CMTPX or CMTMR. The labeled cells were injected into young and aged hosts that were treated 15–18 hours earlier with 25 μg of FITC-labeled anti-gp38 antibody. Host mice were euthanized and intact inguinal LN was explanted and visualized using multi-photon excitation microscopy as previously described^37^.

### Data Analysis

Flow cytometry data was analyzed using FlowJo software (TreeStar). Graphing and statistical analysis were performed using Prism 5 software (GraphPad).

## Additional Information

**How to cite this article**: Becklund, B. R. *et al.* The aged lymphoid tissue environment fails to support naïve T cell homeostasis. *Sci. Rep.* **6**, 30842; doi: 10.1038/srep30842 (2016).

## Supplementary Material

Supplementary Information
