# Supplementary material for: The aged lymphoid tissue environment fails to support naïve T cell homeostasis
Source: Sci Rep. 2016 Aug 2;6:30842. doi: 10.1038/srep30842 (PMC4969611; doi:10.1038/srep30842)

The aged lymphoid tissue environment fails to support naïve T cell homeostasis

Bryan R. Becklund[1,2], Jared F. Purton[2], Chris Ramsey[2], Stéphanie Favre[3], Tobias K. Vogt[3], Christopher E. Martin[1,2], Darina S. Spasova[1,2], Gor Sarkisyan[2], Eric LeRoy[2], Joyce T. Tan[2], Heidi Wahlus[2], Brea Bondi-Boyd[2], Sanjiv A. Luther[3] and Charles D. Surh*[1,2,4,5]

[1]Department of Developmental Immunology, La Jolla Institute for Allergy and Immunology, La Jolla, CA, 92037, USA

[2]Department of Immunology and Microbial Science, The Scripps Research Institute, La Jolla, CA, 92037, USA

[3]Department of Biochemistry, Center for Immunity and Infection, University of Lausanne, 1066 Epalinges, Switzerland

[4]Academy of Immunology and Microbiology, Institute for Basic Science, Pohang, Republic of Korea

[5]Department of Integrative Biosciences and Biotechnology. Pohang University of Science and Technology, Pohang, Republic of Korea

***Corresponding Author:** Charles D. Surh, Academy of Immunology and Microbiology, Institute for Basic Science, Pohang 790-784, Republic of Korea; csurh@ibs.re.kr

Supplementary Figure 1: The number of naïve T cells declines in aged mice. (**A-C**) Cells isolated from the pLN, mLN, and spleen of young and aged B6.Foxp3^{GFP+} mice were stained with anti-TCR-β, CD4, CD8, and CD44 antibodies and the number and phenotype of T cells was determined by FACS. **A**) Representative histograms show CD44 staining gated on TCR-β^{+} Foxp3^{GFP-} CD4^{+} or CD8^{+} T cells in young and aged mice. Numbers indicate the percentage of CD44^{hi} MP CD4^{+} or CD8^{+} T cells. **B**) Bars demonstrate the average number of naïve (CD44^{low}) or MP (CD44^{high}) T cells (±SD) recovered from young and aged mice (n=3 mice per group). **C**) Bars depict the recovery of naïve CD4^{+} or CD8^{+} T cells (±SD) from young and aged mice. *P<0.05, **P<0.01, and ***P<0.001 as determined by unpaired Student's T-test.

Supplementary Figure 2: Reduced T cell homeostatic proliferation in pLN, mLN, and spleen. (**A-B**) Naïve T cells from young B6.CD45.1^{+} mice were labeled with CTV and 1.5 X 10^{6} T cells were injected into irradiated young and aged B6.CD45.2^{+} hosts. Hosts were injected i.p. with 3 mg/kg FTY720 on 1 and 3 days after cell transfer. Proliferation of donor cells was analyzed 7 days after adoptive transfer. Histograms show the proliferation of CD4^{+} (**A**) or CD8^{+} (**B**) donor T cells in the indicated tissue of young and aged hosts. Histograms are representative of 2 independent experiments with 3-4 mice per group.

Supplementary Figure 3: Signaling defects in the mLN and spleen of aged mice. (**A-B**) Naïve T cells from young B6.CD45.1^{+} mice were injected into irradiated young (2 mo) and aged (22 mo) B6.CD45.2^{+} hosts. Phosphorylation of STAT5 (**A**) and CD127 expression (**B**) were analyzed in the

mLN and spleen one day later by flow cytometry. (**C**) Sorted MP $CD4^+$ and $CD8^+$ T cells were injected into irradiated young (2 mo) and aged (18 mo) hosts. One day later, donor cell pSTAT5 and CD127 levels were analyzed in the pLN by flow cytometry. Data is representative of 2 independent experiments with 2-5 mice per group. *P<0.05 and **P<0.01 as determined by unpaired Student's T-test.

Supplemental Figure 1:

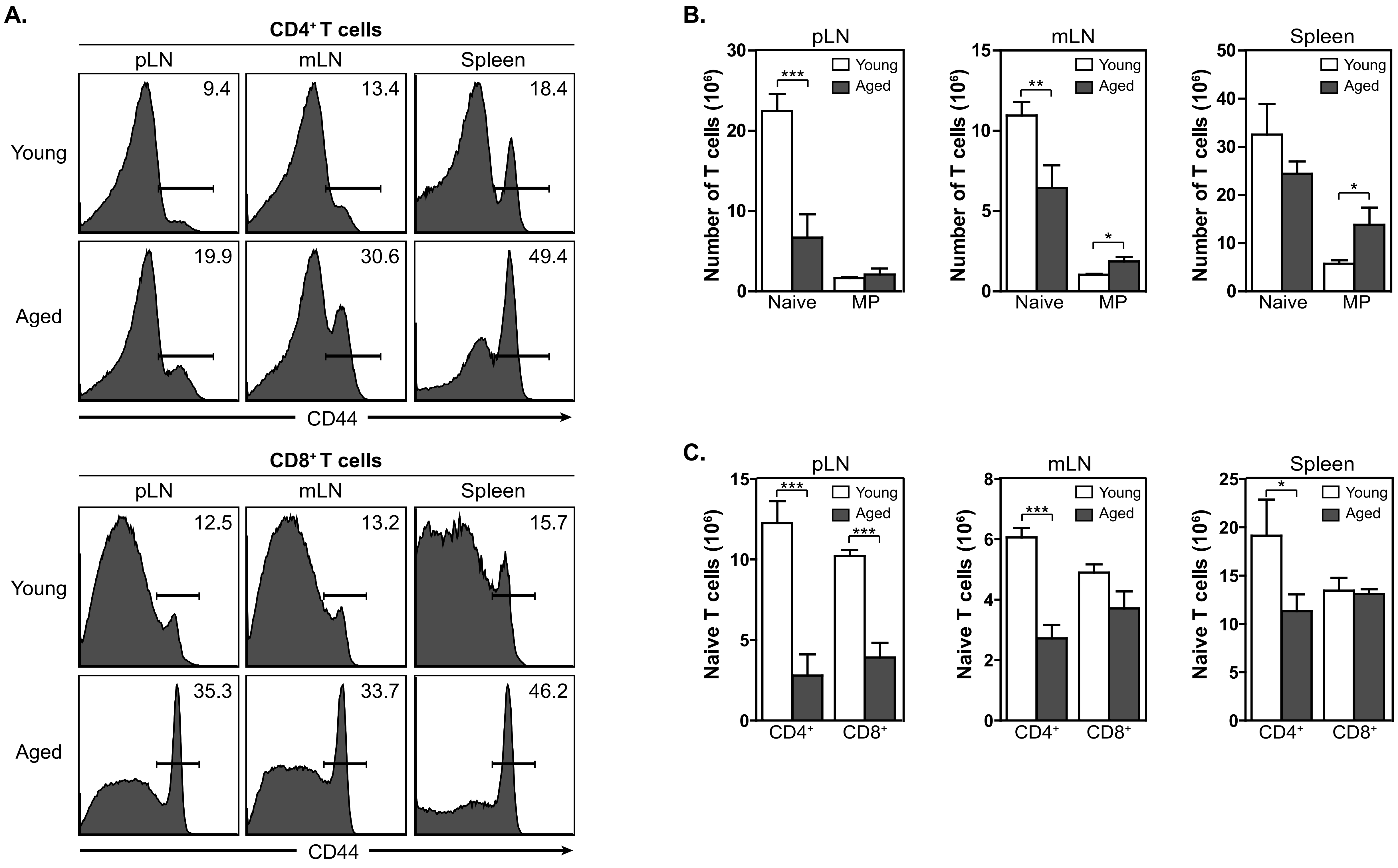

Supplemental Figure 2:

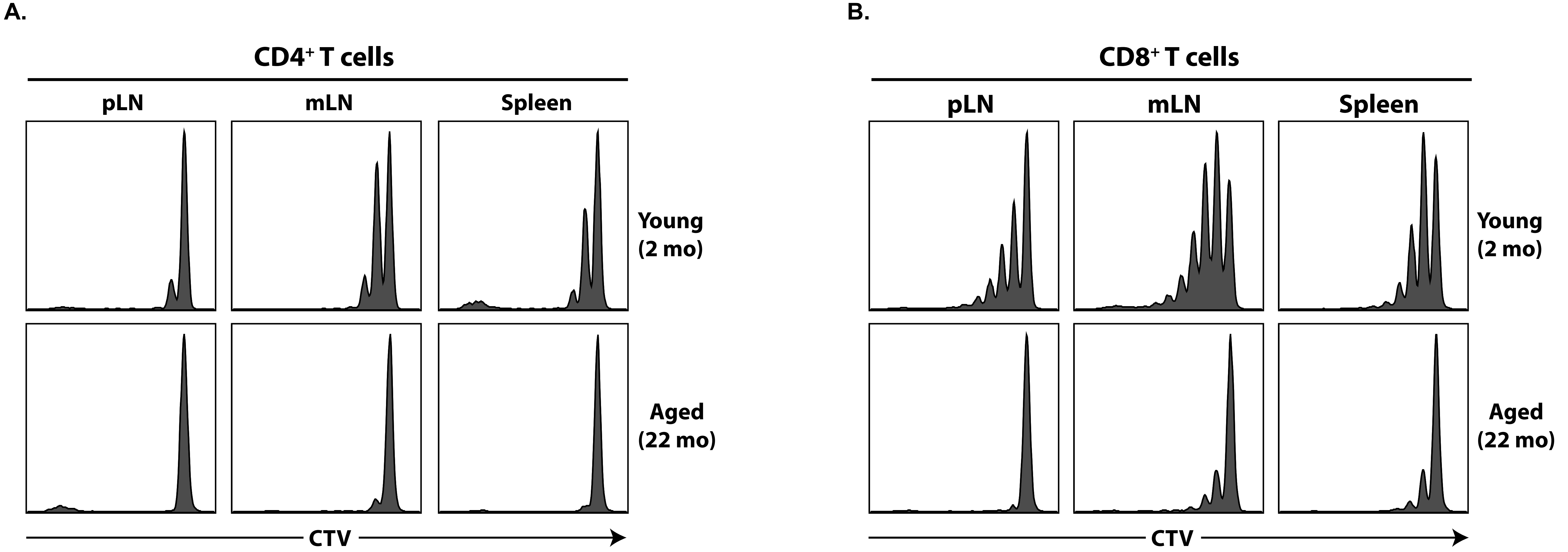

Supplemental Figure 3:

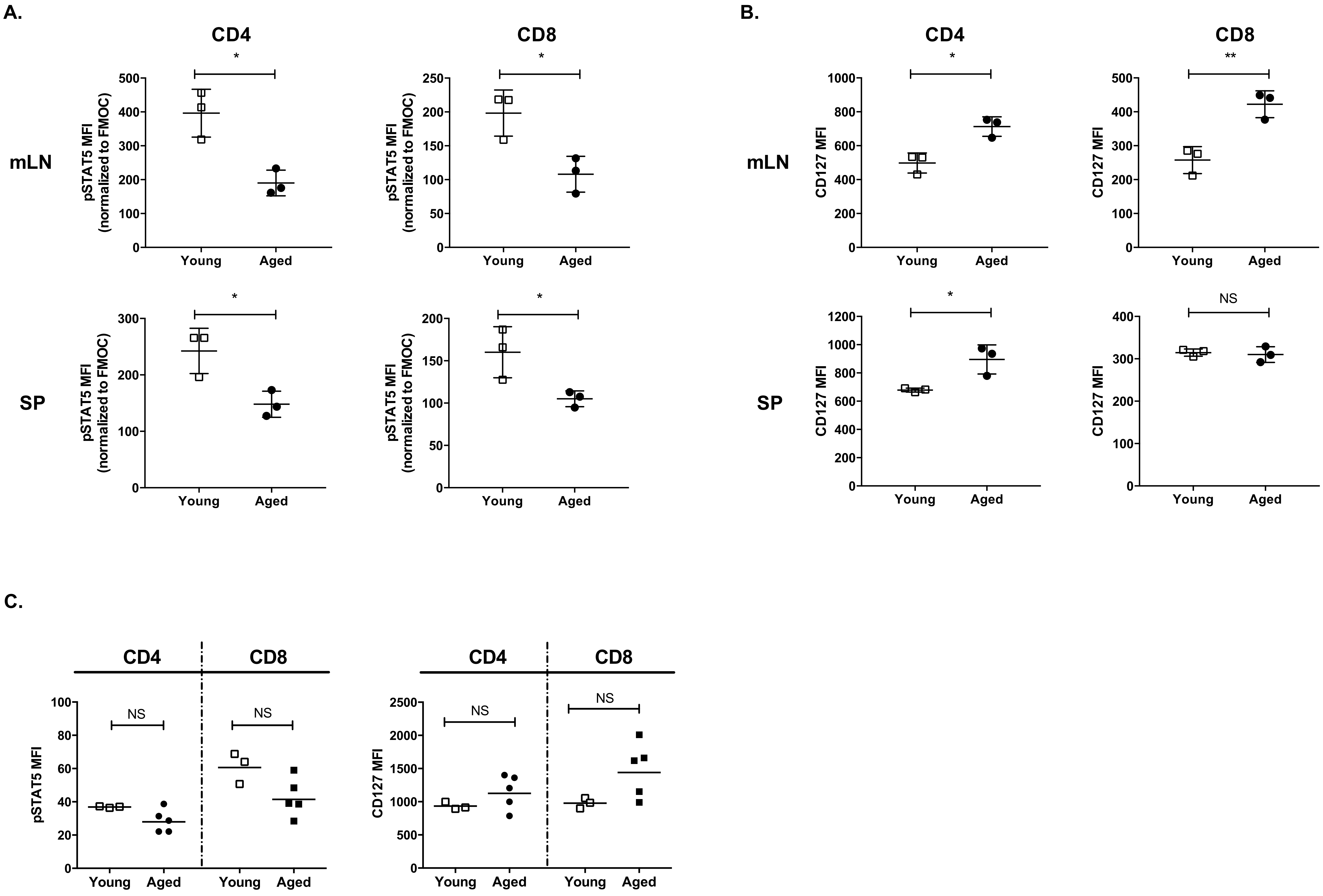

Supplement: Supplementary Information [file srep30842-s1.pdf]
